# Swallow Syncope Associated With Intermittent Sinus Pause and High-Degree Atrioventricular Block: A Case Report

**DOI:** 10.7759/cureus.22915

**Published:** 2022-03-07

**Authors:** Kevin Malone, Malak Modi, Sandeep Koripalli, Allen Amorn, Christopher M Stevens

**Affiliations:** 1 Emergency Medicine, Louisiana State University Health Sciences Center, Shreveport, USA; 2 Internal Medicine, Louisiana State University Health Sciences Center, Shreveport, USA; 3 Internal Medicine, St. Barnabas Hospital, Bronx, USA; 4 Cardiology, Louisiana State University Health Sciences Center, Shreveport, USA; 5 Interventional Radiology, Louisiana State University Health Sciences Center, Shreveport, USA

**Keywords:** block, atrioventricular, bradycardia, syncope, swallow

## Abstract

Swallow syncope, also known as deglutition syncope, is a relatively rare neurogenic disorder that is triggered by oral intake. When diagnosed, swallow syncope is treatable, but it is challenging to identify without proper history and prior knowledge of this disorder. Here, we describe the case of a 68-year-old female with a complex history who presented to the emergency room with complaints of worsening long-term intermittent lightheadedness and dizziness associated with swallowing. During her hospital stay, the patient was noted to have a high-degree atrioventricular block on telemetry during dinner time. A repeat electrocardiogram (ECG) demonstrated a prolonged P-R interval, and a temporal relationship between swallowing cold water and electrocardiogram (ECG) changes was demonstrated. A diagnosis of swallow syncope was confirmed. Electrophysiology was consulted and a pacemaker was placed. Symptoms of swallow-associated arrhythmias completely resolved after pacemaker implantation. This case illustrates the challenging problem of swallow syncope. While swallow syncope is highly treatable once identified, the challenge lies in identifying the disorder.

## Introduction

Swallow syncope is a rare type of neurogenic-mediated syncope associated with life-threatening bradyarrhythmia and hypotension. To date, there have been fewer than 150 cases recorded in the literature [1,2]. The pathophysiology of swallow syncope is not completely understood, and several different mechanisms causing it may have not yet been discovered [3]. Swallow syncope is a dysautonomic syndrome associated with vagal afferent activation caused by esophageal stimulation, meaning that it is a dysfunction of the vagal nerve that regulates the heart, causing arrhythmia when swallowing [4]. In some cases, it may cause complete loss of consciousness through the reduction of blood flow to the brain caused by arrhythmia [4].

Mechanoreceptors in the esophagus, which are activated by stretching or movement, possibly play an important role [5]. The esophagus and the heart share the same innervation by the vagus nerve. Stretching of the esophagus through the act of swallowing sends afferent signals along the esophageal plexus, via the left vagus nerve, to the brainstem. The efferent impulses from the brainstem may then reach the sinoatrial node via the right vagus nerve and the atrioventricular (AV) node via the left vagus nerve. These efferent signals may lead to several different bradyarrhythmias and temporary reductions in cardiac output. As a result of this decrease in cardiac output, there is ultimately a reduction in cerebral hypoperfusion, leading to syncope [6]. Some of the bradyarrhythmias that have been reported in swallow syncope include sinus bradycardia, sinoatrial block, complete asystole, and, most frequently, AV block. Paroxysmal atrial fibrillation has also been associated with swallow syncope in rare cases [7].

Functional disorders of the esophagus are a common occurrence in swallow syncope; however, they may occur in the absence of such disorders. Disorders of the esophagus that have been described with swallow syncope include esophageal spasm, esophageal stricture, achalasia, esophageal diverticula, esophageal cancer, and hiatal hernia. In some cases, cardiac diseases, such as ascending aortic aneurysm, thoracic surgery, advanced lung cancer, and transient hypoxia, have also been described as associated pathologies with swallow syncope [1,4]. However, in a significant number of cases, there are no underlying causative pathologies described [8]. According to the current literature, most cases of swallow syncope appear in older male adults, less frequently in adult females, and even less in children [5].

## Case presentation

A 68-year-old female was brought to the emergency room with complaints of worsening long-term intermittent lightheadedness and dizziness. She reported that the day before admission, she had a complete loss of consciousness after swallowing a pickle, which was witnessed by her family. The loss of consciousness lasted until paramedics arrived, estimated to be 20 minutes, without any postictal period.

The patient had a history of coronary artery disease with three stents for which she was on beta-blocker medication. The patient had a recent transient ischemic attack two months prior with left-sided weakness, which had completely resolved. She also had a history of a neuroendocrine tumor of the terminal ileum with resection seven years ago, and a history of breast cancer with resection seven months ago. Other medical history included hypertension, diabetes, interstitial lung disease, for which she was on oxygen, and a recently found thyroid nodule.

Physical examination and review of systems were mostly unremarkable other than obesity. At the time of assessment in the hospital, she denied any current complaints. Vitals at examination showed a heart rate of 88 beats per minute, blood pressure of 172/83 mmHg, respiratory rate of 20 breaths per minute, and oxygen saturation of 97% on 2 L of oxygen. Orthostatic vitals were inconsequential. She was given an ABCD2 score of 4.

**Figure 1:**
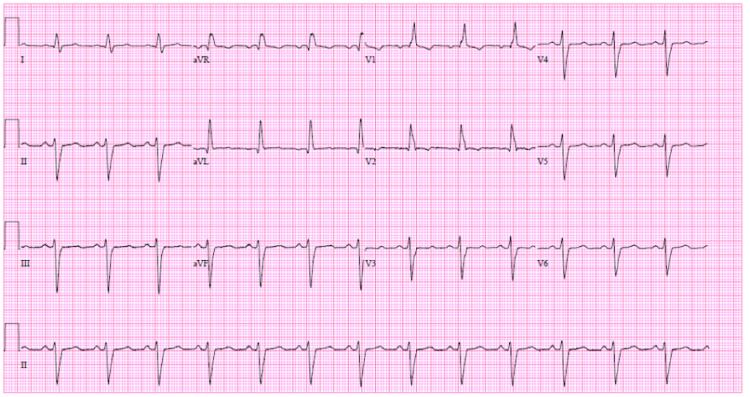
Initial ECG. Initial 12-lead ECG on presentation while the patient was at rest. New bifascicular block compared with previous ECG seven months prior, which showed only a right bundle branch block. ECG: electrocardiogram

Imaging included electrocardiogram (ECG), which when compared to a prior ECG showed a bifascicular block (Figure 1); X-ray of the chest, which showed interstitial lung markings and calcified nodule; computed tomography of the neck soft tissues, which showed bilateral hypodense thyroid nodule and reactive lymph node; ultrasound of thyroid showed Thyroid Imaging Reporting and Data System (TIRADS)-3 on the right and TIRADS-1 on the left, which required no follow-up or fine-needle aspiration; transthoracic echocardiogram with contrast resulted in normal left ventricular diastolic function mild mitral regurgitation and ejection fraction of 60-65%; and an esophagram showed no major deficits.

**Figure 2:**
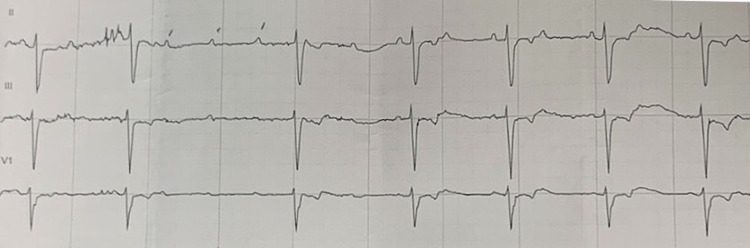
Overnight ECG. Transient high-grade, second-degree AV block on a background of 2:1 second-degree AV block. ECG: electrocardiogram; AV: atrioventricular

Significant lab work included complete blood count and chemistry showed no significant change from baseline; three troponins I, each of which were negative; brain natriuretic peptide of 136 pg/mL; thyroid-stimulating hormone of 2.2 uIU/mL; A1c of 8.3%; and chromogranin significantly elevated to 1,220 ng/mL from baseline (90 ng/mL) post-surgical resection. After the initial ECG, the patient was monitored overnight while on telemetry (Figure 2).

Telemetry only alerted for bradycardia in the night. Only when the strip was personally examined, it was noted to have a high-grade, second-degree AV block with three consecutively blocked P waves. These changes corresponded to hours when dinner was typically served. The patient confirmed that she was eating at this time and stated that she had a brief episode of lightheadedness. A repeat ECG was then obtained in the morning which showed no severe abnormalities. Beta-blocker medications were subsequently discontinued.

On further clarification, the worsening long-term intermittent lightheadedness and dizziness were also associated with swallowing. Her symptoms of lightheadedness and dizziness were almost always associated with swallowing and were usually resolved within an hour after swallowing. She was unsure of the duration of swallowing difficulties, only stating that it had been “multiple years.” She stated that she had been cutting her food into very small pieces to prevent the syncopal episodes, which she said had improved in the past. She stated that she had been evaluated for swallowing difficulties in the past which resulted in no definitive diagnosis.

**Figure 3:**
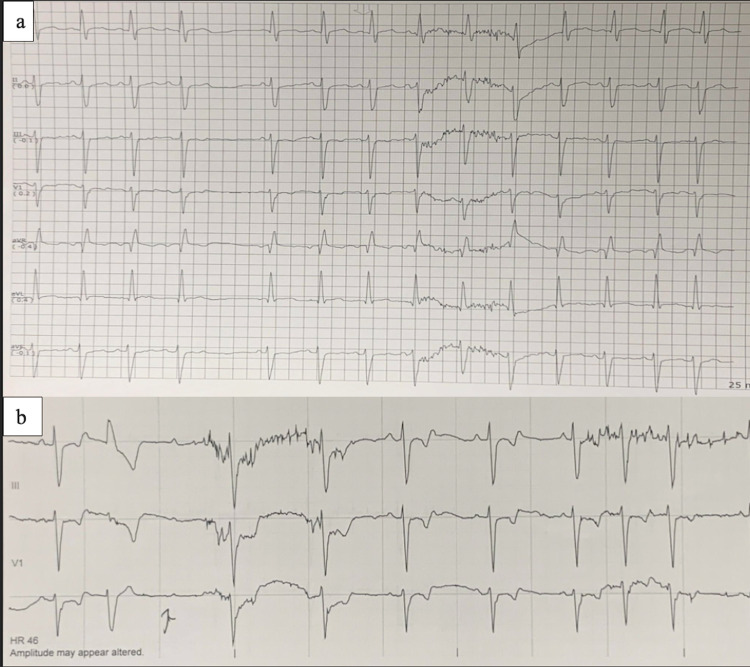
Provocative testing. (a) Sinus arrest five seconds after the initiation of drinking cold water. (b) AV block 10 seconds after swallowing cold water. AV: atrioventricular

Provocative testing by requesting the patient to drink cold water, while under telemonitoring, was attempted to confirm a possible diagnosis of swallow syncope (Figure 3).

The patient also reported dizziness and light-headedness immediately after swallowing the cold water. Positive demonstration of a temporal relationship between swallowing, symptoms of light-headedness, dizziness, and ECG changes was confirmed. A diagnosis of swallow syncope was given. Electrophysiology was consulted, and the patient was given a permanent pacemaker dual-chamber, rate-modulated type. The patient had complete resolution of all symptoms. A timeline of events is listed in Table 1.

**Table 1:** Timeline of events. AV: atrioventricular

Sequential number	Action/Event
1	The patient presents to the emergency department for a syncope-like episode
2	Admitted to the hospital; no absolute causative factors were found initially
3	While on telemetry, high-degree, AV block was seen associated with the patient’s mealtime
4	Clarification of the patient’s symptoms reveals temporal relationship of syncope with swallowing
5	Provocative testing demonstrated temporal relation of heart block with swallowing
6	Diagnosis confirmed, and pacemaker placed
7	The patient had complete resolution of symptoms

## Discussion

This diagnosis of swallow syncope may often be missed because of poor history taking or due to a lack of prior knowledge regarding this rare disorder. Further, there may be a complete absence of signs or symptoms in many cases unless the patient is actively swallowing. Therefore, careful history taking is necessary to recognize the temporal connection between swallowing and syncopal symptomatology. Telemetry monitoring and provocation tests may reveal a correlation between swallowing and cardiac or syncope episodes. Provocative testing with various liquids and solid foods should be attempted. Asking the patient which foods or liquids elicited the syncope response in the past and duplicating the prior situation under telemetry or continuous ECG monitoring may provide a definitive diagnosis.

A previous study evaluated heart rate and hemodynamic changes in patients affected with swallow syncope versus asymptomatic patients. In the study, a greater decrease in both heart rate (-22 ± 22.1 vs. -3 ± 11.7 beats/minute; P = 0.045) and blood pressure (-22 ± 17.4 vs. -2 ± 11.8; P = 0.036) was found in swallow syncope patients when compared with controls [9]. Additionally, in the affected group, it was found that the time to lowest heart rate and blood pressure differed (9 ± 5.5 vs. 19 ± 7.2 seconds; P = 0.02), which suggests that both vasodepressor and cardioinhibitory mechanisms were present and operating independently [9]. Further studies to exclude the possible structural or functional esophageal pathology have also been suggested. A baseline ECG and echocardiogram might help to exclude an underlying cardiac pathology, although the lack of deficits may not exclude a diagnosis. An esophagogastroscopy or barium study can be ordered to detect an underlying disease of the esophagus; however, the absence of pathology does not necessarily rule out the diagnosis [10].

Withdrawal of all medications that may cause a delay in cardiac conduction and inappropriate vasodepression should be initiated first. Permanent pacemaker insertion has become the first-line treatment in recent years. It is especially useful for patients suffering from cardioinhibition with swallowing or without correctable esophageal pathology. Surgical correction of esophageal pathology has been successful in some cases if it is believed to be of esophageal origin [11].

Medications that affect vagal modulation, such as atropine, may be another choice in management. In the past, some medications, such as epinephrine or isoprenaline, have been used to increase the ventricular rate directly [12,13]. No pharmacological interventions have been uniformly successful, and most medications are poorly tolerated. In some cases, diet changes, such as avoiding carbonated fluids and excessively hot or cold liquids, reduced stimulation of the esophagus, suggesting that correction of eating habits may be all that is necessary for treatment [14].

## Conclusions

This case illustrates the challenging problem of swallow syncope. Although swallow syncope is highly treatable once identified, the challenge lies in identifying this rare disorder. Swallow syncope should be considered a potential diagnosis for patients presenting with syncope or near syncope. Patient history is especially important in demonstrating a temporal relationship for this diagnosis. Provocative testing, that is, having the patient swallow while observing telemetry, can help establish the diagnosis. A baseline ECG and echocardiogram might help to exclude underlying cardiac pathology. Permanent pacemaker implantation is generally the first-line curative treatment.
